# Ranolazine improved left ventricular diastolic functions and ventricular repolarization indexes in patients with coronary slow flow

**DOI:** 10.3389/fcvm.2023.1207580

**Published:** 2023-08-21

**Authors:** Dogac Oksen, Muzaffer Aslan, Emre Ozmen, Yunus Emre Yavuz

**Affiliations:** ^1^ Department of Cardiology, Faculty of Medicine, Altınbaş University, Istanbul, Türkiye; ^2^ Department of Cardiology, Faculty of Medicine, Siirt University, Siirt, Türkiye

**Keywords:** ranolazine, repolarization indexes, coronary slow flow, tissue doppler imaging, coronary circulation, electrocardiography

## Abstract

**Introduction:**

Coronary slow flow (CSF) is a condition commonly encountered during angiography. Recent studies have shown the adverse effects of CSF on left ventricular diastolic functions. CSF reportedly increases the novel ventricular repolarization parameters. Ranolazine is a preparation with a prominent anti-anginal activity that has positive effects on anti-arrhythmic and diastolic parameters. In this context, this study was carried out to investigate the effects of ranolazine on left ventricular diastolic functions and repolarization in patients with CSF.

**Material and methods:**

Forty-six patients with CSF and 29 control subjects were included in the patient and control groups, respectively. Both groups received ranolazine for one month and were evaluated using 12–lead electrocardiography, conventional echocardiography, and tissue Doppler imaging at the baseline and after one month of ranolazine treatment.

**Results:**

Corrected P, QT dispersion, and Tp-e interval values were significantly higher in the patient group than in the control group. There was a significant decrease in isovolumic relaxation time (IVRT) and deceleration time (DT) values after the ranolazine treatment compared to the baseline values in the patient group but not the control group. A significant increase was observed in the mean E and A velocities and the mean E/A ratio after the ranolazine treatment compared to the baseline values in the patient group. Additionally, there was a significant difference between the Tp-e interval and corrected *P* dispersion values measured after the ranolazine treatment compared to the baseline values in the patient group but not in the control group.

**Conclusion:**

This study's findings demonstrated that ranolazine positively affected impaired diastolic functions and repolarization parameters, particularly in patients with CSF.

## 1. Introduction

Coronary artery disease (CAD) is one of the major causes of morbidity and mortality worldwide. Various clinical subgroups of CAD have been defined (1). Coronary slow flow phenomenon (CSF) is currently considered under CAD since it features a slowdown in the rate of progression of opaque material to the distal coronary artery despite the absence of visible atherosclerotic pathology in the coronary arteries (2). CSF is frequently asymptomatic. Nevertheless, CSF patients may occasionally present with stable angina pectoris, acute coronary syndrome, various arrhythmias, and albeit rarely, sudden cardiac death (3). The increase in relevant interventional procedures confuses the interventional cardiologists dealing with this phenomenon. Manifestation, hence the epidemiology, of CSF has not been fully established in the literature, as have the related clinical follow-up and treatment practices are still considering. The clinical features of the CSF have been addressed in many studies to date. The majority of these studies indicated that metabolic syndrome factors are associated with the risk of CSF phenomenon (3).

The prognosis in most CSF patients is reportedly good in terms of cardiovascular events, yet some suffer from persistent chest pain requiring medical therapy. In the chronic coronary syndrome's guidelines of the European Society of Cardiology published in 2019, CSF was not categorized as a separate group but under the microvascular diseases subgroup with relevant treatment recommendations (1). Beta-blockers and calcium channel blockers are the primarily recommended medications for the treatment of angina in non-obstructive CAD, even though further evidence is needed to demonstrate their efficacy in this patient group.

Ranolazine is a piperazine derivative drug that inhibits late sodium currents. It is a second-line therapeutic agent in managing chronic stable angina pectoris. Ranolazine is reportedly effective on arrhythmias in addition to angina. Ranolazine counteracts the increase in intracellular calcium by inhibiting the late sodium currents and facilitating the relaxation of myocardial cells. Additionally, it promotes myocardial electrical stability by preventing intracellular calcium overload, which is a predisposing factor for ventricular tachycardia (4).

T wave on electrocardiography (ECG) indicates repolarization and is used as a marker of dispersion of ventricular repolarization. Previous studies demonstrated that ventricular repolarization parameters predictive of ventricular arrhythmias, such as Tp-e interval, Tp-e/QT ratio, and Tp-e/QTc, are affected in CSF (5). Studies evaluating CSF via echocardiography have reported impaired left ventricular diastolic functions. Except for obstructive CAD, the pathogenesis of diastolic dysfunction in CSF has not been fully elucidated. It is known that the diastolic functions of patients with acute coronary syndrome improve after treating the underlying ischemia (6). In this context, this study was carried out to investigate the effects of ranolazine on left ventricular repolarization and diastolic function parameters in patients with CSF.

## 2. Material and method

Forty-six CSF patients admitted to the outpatient cardiology clinic with chest pain and underwent coronary catheterization between April 2021 and June 2022, and 29 control subjects with normal coronary artery findings in coronary angiography were included in the patient and control groups, respectively. The exclusion criteria were having left ventricular ejection fraction (LVEF) < 50%, coronary ectasia, coronary artery plaque formation, coronary artery disease, moderate-to-severe valvular regurgitation or stenosis, hyperthyroidism, chronic obstructive pulmonary disease, myocardial infarction history, an implanted pacemaker or defibrillator, bundle branch block, any kind of rhythm except sinus rhythm, ventricular preexcitation, and prolonged QT. A 12-lead ECG and transthoracic echocardiography (TTE) were performed initially on all patients who underwent coronary angiography via radial or femoral access. The patient and control groups received 500 mg of ranolazine twice daily for one month. Patients were re-evaluated by ECG and TTE after six weeks from the start of the treatment. The study protocol was approved by the ethics committee of Siirt University Faculty of Medicine. The study was carried out in accordance with the ethical principles set forth in the Declaration of Helsinki and Good Clinical Practice Guidelines. Informed consent was obtained from all participants included in the study.

### 2.1. Assessment of CSF and thrombolysis in myocardial infarction frame count

Coronary angiographies of the patients were performed with Siemens Artis Zee Floor angiography device (Siemens, Erlangen, Germany) using the standard Judkins technique. Iohexol 350/100 ml was used as the radio-opaque material during the procedure, and the contrast material was manually injected 5–6 ml per cine. The cinefilming rate of angiography was 30 frames/s. CSF was diagnosed based on the definition of thrombolysis in myocardial infarction frame (TIMI) count defined by Gibson et al. (7).

After administering the radio-opaque material, the number of frames during the arrival of the opaque to the distal portion of the coronary arteries was calculated separately for each of the three coronary arteries, which were then averaged. The most distal bifurcation alignment was determined for the left anterior descending artery (LAD) and circumflex (Cx) as distal edge, and the alignment of the first branch of the posterolateral artery was determined for the right coronary artery (RCA). Since the LAD artery was longer than RCA and Cx, the frame number of LAD was divided by 1.7 for adjustment. TIMI frame count evaluation was performed from the right caudal position for LAD and Cx and the left cranial position for RCA (8). The angiographic records were evaluated by two independent investigators blinded to the groups in order to avoid bias.

### 2.2. Echocardiographic assessment

Two proficient researchers assessed the participants using a GE Vivid S6 echocardiography system (Horten, Norway) with 3 and 7.5 MHz transducers. The baseline characteristics and demographic data of the participants were documented. The left lateral decubitus position was utilized throughout the assessments, and continuous telemetric monitoring was performed using a single-lead ECG. The average of three consecutive contractions was recorded. During these assessments, parasternal, apical, and sub-costal views were scrutinized. From the four-chamber apical view, E and A mitral inflow velocities and the respective E/A ratio were determined using pulse-wave (PW) Doppler Imaging. During the diastolic phase, the Doppler tip was positioned over the mitral valve leaflets. The dimensions of the left atrium (LA), right atrium (RA), and end-diastolic and end-systolic measurements of the left ventricle (LV) were measured from the parasternal long-axis view. The baseline dimension of the right ventricle was determined from the apical four-chamber view. The biplane-modified Simpson technique was employed to ascertain LA volumes. LA measurements were performed at LV end-systole from both apical 4-chamber and 2-chamber views. The ejection fraction of the left ventricle was calculated using Simpson's method. All measurements were performed according to the American Society of Echocardiography and European Association of Cardiovascular Imaging guidelines (7).

### 2.3. Electrocardiography

Participants underwent 12-lead surface ECG (Nihon Kohden, Tokyo, Japan) at 10 mm/mV amplitude and 25 mm/s rate while at rest and in the supine position. The QT and Tp-e intervals were calculated manually by using a caliper. Precordial leads were used to assess the Tp-e interval. The Tp-e interval was calculated as the time from the peak of the T wave to the point where the T wave joins with the isoelectric line and ends. The QT interval was defined as the duration from the QRS complex to the end of the T wave. Corrected QT interval corrected for the heart rate (QTc) was calculated according to Bazett's formula (9). The QT dispersion was calculated as the difference between the twelve leads' longest and shortest QT intervals. RR interval was considered the average time elapsed between three successive R-waves of the QRS signal. The variation coefficients of interobserver and intraobserver agreements were 2.9% and 3.3%, respectively.

### 2.4. Statistical analysis

SPSS 23.0 (Statistical Product and Service Solutions for Windows, Version 23.0, IBM Corp., Armonk, NY, U.S., 2015) software package was used in the statistical analyses of the collected data. Continuous variables were expressed as mean ± standard deviation values, whereas categorical variables were expressed as percentage values. Non-normally distributed variables were expressed as median with minimum-maximum values. The distribution characteristics of the variables were analyzed by Kolmogorov-Smirnov and Shapiro-Wilk tests. For quantitative and parametric variables Student—T test was used. Non- parametric quantitative data were assessed by Mann–Whitney' s *U* test. The relationship between qualitative variables chi- square test was used. Friedman test was used when the assumption of normality was not provided in the dependent groups. The paired sample *t*-test was used to evaluate the differences between continuous variables. Pearson's correlation coefficients were used to assess intra-observer and inter-observer agreements. Probability (*p*) values less than 0.05 were deemed to indicate statistical significance.

## 3. Results

A total of 75 participants, 46 in the patient and 29 in the control groups were included in the study. The mean age of the patient and control groups was 55.22 ± 9.33 years and 53.18 ± 8.32 years, respectively. In terms of gender distribution, the ratio of males was 60.9% (*n* = 28) and 44.8% (*n* = 13) in the patient and control groups, respectively. There was no significant difference between the groups in demographic characteristics, vital findings, and baseline echocardiographic data (Table 1). Isovolumetric relaxation time (IVRT) was significantly longer in the patient group than in the control group (87.85 ± 18.67 ms vs. 78.21 ± 9.86 ms; *p* = 0.013). Although there was no significant difference between E and A waves in the patient and control groups, E/A was significantly higher in the control group than in the patient group (1.02 ± 0.26 vs. 0.96 ± 0.28; *p* = 0.038). There was no significant difference between the groups in terms of *P* dispersion, QT dispersion, and Tp-e interval. However, the corrected values of these parameters according to the heart rate, i.e., corrected *P* dispersion, QTc dispersion, and corrected Tp-e interval, were significantly higher in the patient group than in the control group. In addition, the TIMI frame count of all three coronary arteries (LAD, Cx, and RCA) and the mean TIMI frame count were significantly higher in the patient group than in the control group.

**Table 1:** Clinical characteristics, ECG and echocardiographic findings.

Variables	CSF (*n* = 46)	Control group (*n* = 29)	*p*-value
Age, years	55.22 ± 9.33	53.18 ± 8.32	0.104
Sex (Male), %(*n*)	60.9% (28)	44.8% (13)	0.173
BMI, kg/m^2^	29.79 ± 4.17	30.65 ± 4.47	0.403
DM, %(*n*)	45.7% (21)	44.8% (13)	0.944
HT, %(*n*)	56.5% (26)	41.4% (12)	0.201
Smoker, %(*n*)	45.7% (21)	31% (9)	0.208
Heart Rate, beats/min	75.80 ± 12.60	76.24 ± 12.72	0.885
SBP, mmHg	127.67 ± 13.51	128.66 ± 12.38	0.753
DBP, mmHg	78.83 ± 8.12	79.03 ± 6.58	0.908
LVED diameter**, mm	48 (46–51)	47.55 ± 2.84	0.173
LVES diameter**, mm	28 (25–28)	28.28 ± 3.29	0.631
LVEF**, %	64.20 (61.57–65.62)	63.05 ± 3.09	0.597
SW, mm	10.80 ± 1.55	10.74 ± 1.24	0.844
PW, mm	10.41 ± 1.24	10.13 ± 1.04	0.313
IVRT, ms	87.85 ± 18.67	78.21 ± 9.86	0.013
Mitral inflow peak E velocity (m/s)	73.21 ± 16.86	75.33 ± 14.71	0.580
Mitral inflow peak A velocity (m/s)	77.98 ± 20.83	76.37 ± 14.22	0.717
E/A	0.96 ± 0.28	1.02 ± 0.26	0.038
DT	201.22 ± 34.16	206.90 ± 27.62	0.454
MeanEm (cm/s)	8.22 ± 1.42	8.40 ± 1.37	0.581
MeanAm (cm/s)	10.64 ± 2.89	10.59 ± 2.30	0.944
MeanEm/Am ratio	0.82 ± 0.27	0.87 ± 0.27	0.013
E/E′ (Em)	9.18 ± 2.34	8.88 ± 2.36	0.589
QT, ms	376.76 ± 20.92	371.31 ± 20.19	0.269
Cor. *P* dispersion	40.90 ± 10.13	37.40 ± 7.89	0.025
Cor. QT dispersion	41.64 ± 7.72	36.49 ± 9.23	0.001
Cor. Tp-e interval	81.52 ± 12.92	76.06 ± 8.69	0.008
Tp-e/QT ratio	0.19 ± 0.02	0.16 ± 0.02	0.016
Tp-e/cQT ratio	0.17 ± 0.22	0.17 ± 0.23	0.808
RR interval	0.81 ± 0.12	0.83 ± 0.14	0.696
TIMI frame count (frame/s)			
LAD	35.00 ± 15.46	16.18 ± 1.38	<0.001
Cx	28.54 ± 11.43	17.84 ± 1.19	<0.001
RCA	34.13 ± 14.49	17.35 ± 1.81	<0.001
Mean TIMI frame count	32.75 ± 11.71	17.11 ± 0.72	<0.001

SBP, systolic blood pressure; DBP, diastolic blood pressure; SW, septal wall; PW, posterior wall; LVED left ventricular end diastol; LVES, left ventricular end systole; A, mitral inflow contraction velocity; Am, atrial contraction wave using TDI; CDI, conventional Doppler imaging; CSF, coronary slow flow; DT, deceleration time; E, mitral velocity of early diastolic filling from transmitral flow; Em, early diastolic filling using TDI; IVRT, isovolumic relaxation time; LV, left ventricle; TDI, tissue Doppler imaging; TIMI, thrombolysis in myocardial infarction; Tp-e, between the peak and the end of the T wave.

Data are presented as mean ± SD and numbers (%).

* Student—T test was used for normally distributed variables.

** Non–normally distributed variables were presented as median (minimum-maximum) value.

** Non–normally distributed variables were assessed by Mann–Whitney’ s *U* test.

The echocardiographic examination performed after administration of twice daily 500 mg ranolazine for one month revealed a significant decrease in IVRT in the patient group but not in the control group (87.85 ± 18.67 vs. 82.24 ± 17.77; *p* = 0.005). Similarly, a significant decrease was observed after the ranolazine treatment in the patient group but not in the control group (201.22 ± 34.16 vs. 189.59 ± 26.13, *p* = <0.001; 206.90 ± 27.62 vs. 204.45 ± 30.47, *p* = 0.338, respectively). There was an increase in mean E and A waves after ranolazine treatment in both groups, yet this increase reached statistical significance only in the patient group (8.22 ± 1.42 vs. 8.49 ± 1.24; *p* = 0.004). Similarly, there was an increase in mean E/A in both groups after ranolazine treatment; however, this increase reached statistical significance only in the patient group (0.82 ± 0.27 vs. 0.90 ± 0.34; *p* = 0.008).

**Figure 1:**
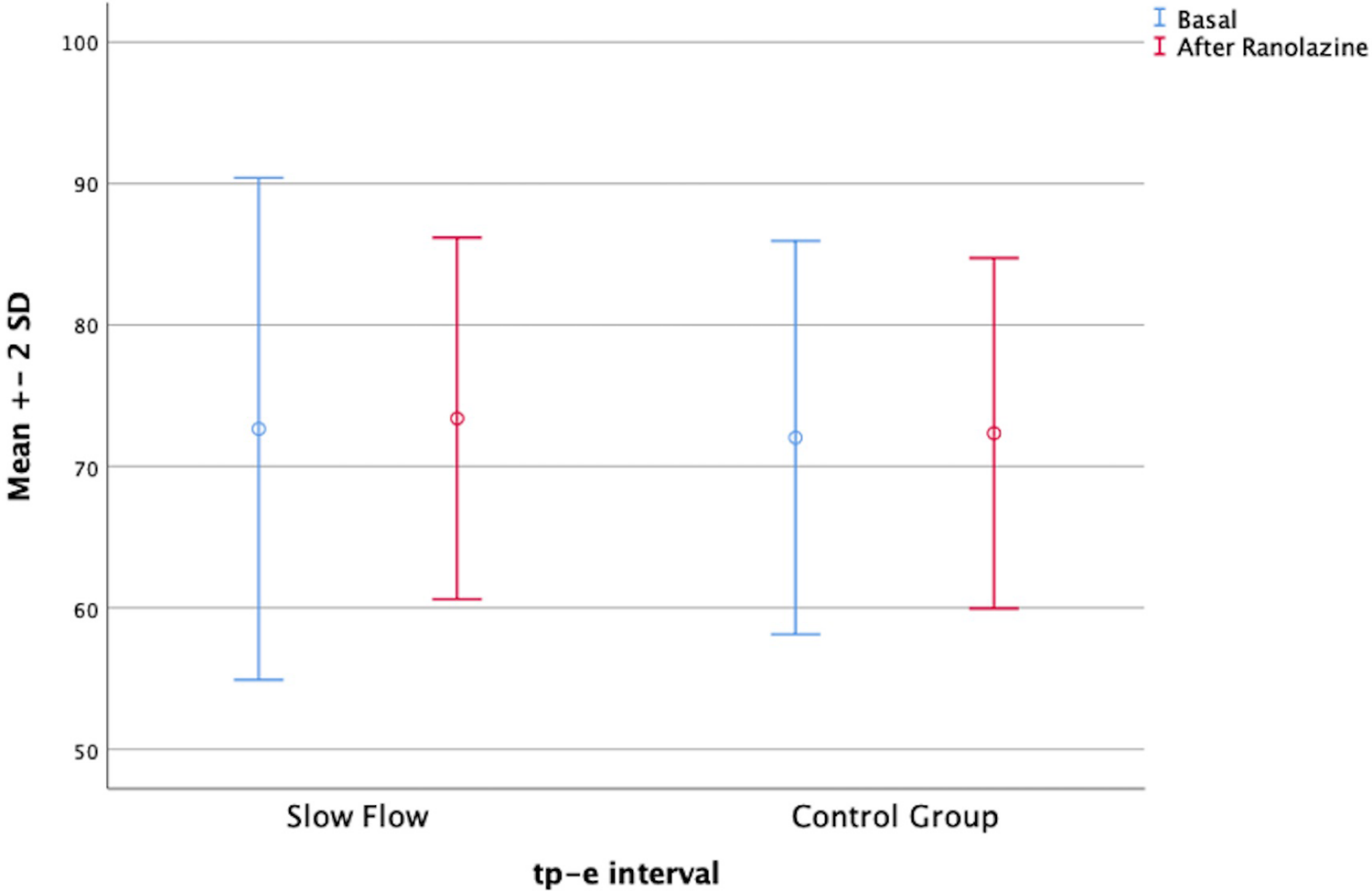
Graphic showing the mean Tp-e interval before and after treatment.

**Figure 2:**
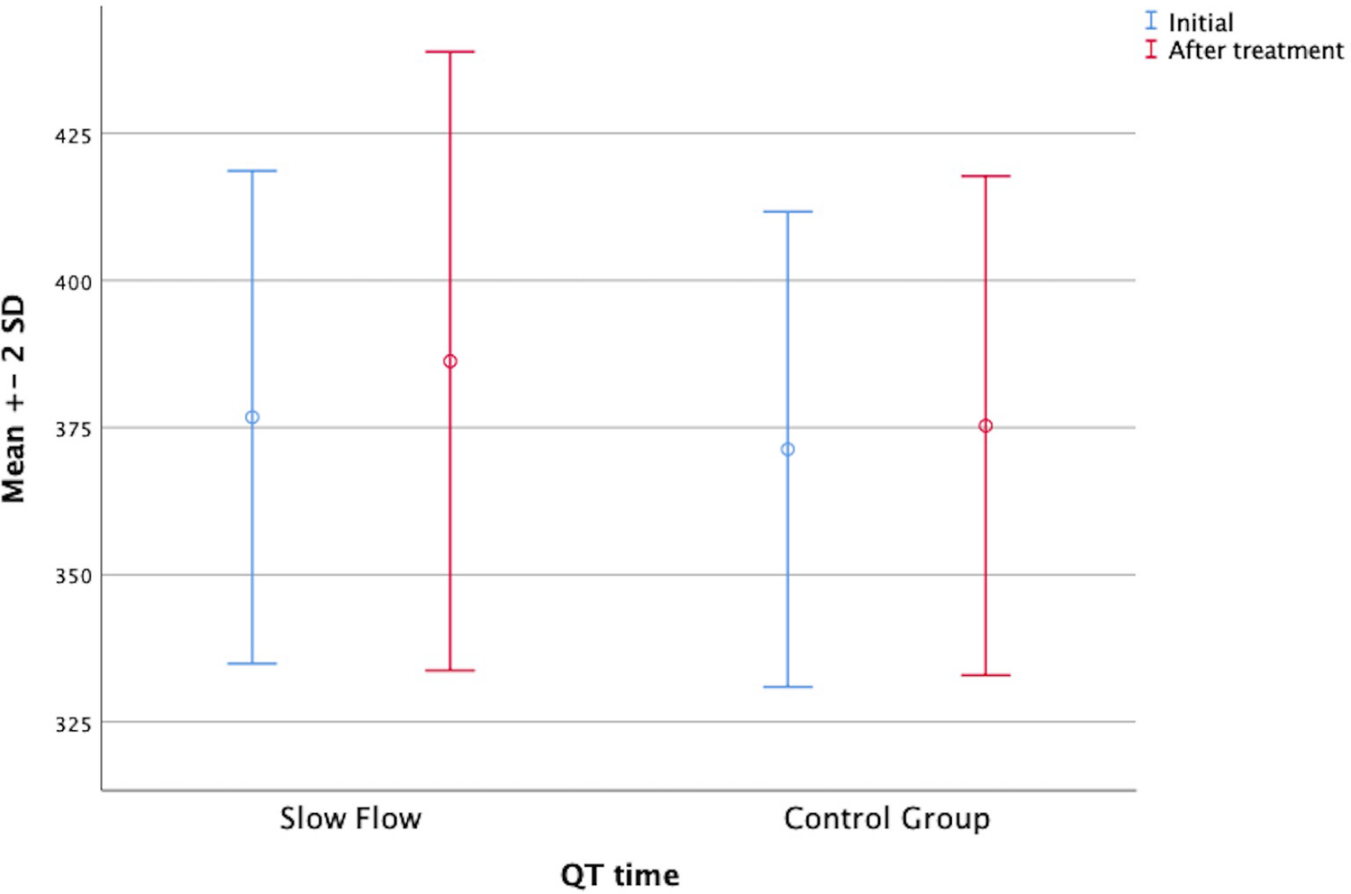
Graphic showing the mean QT interval before and after treatment.

In parallel, the Tp-e interval, the corrected *P* dispersion, and the corrected Tp-e interval were decreased in both groups after ranolazine treatment. However, these decreases were statistically significant only in the patient group (Figure 1). On the other hand, the QT interval was significantly increased in both groups after ranolazine treatment (376.76 ± 20.92 vs. 386.28 ± 26.27, *p* = 0.037; 371.31 ± 20.19 vs. 375.31 ± 21.21, *p* = 0.014) (Figure 2). RR interval increased slightly in both groups, yet this increase reached statistical significance only in the control group, even though the difference between the respective mean values was not too large (*p* = 0.009 for the control group, *p* = 0.431 for the patient group). The distribution of the baseline and post-treatment electrocardiographic and echocardiographic parameters by the patient and control groups is shown in Table 2.

**Table 2:** Echocardiographic parameters and repolarization indexes initial and after ranolazine treatment.

Variables	CSF			Control group		
	Baseline	Follow- up	*p*-value	Baseline	Follow- up	*p*-value
IVRT	87.85 ± 18.67	82.24 ± 17.77	0.005	78.21± 9.86	78.52 ± 9.13	0.734
E′	73.21 ± 16.86	75.66 ± 14.62	0.269	75.33 ± 14.71	75.17 ± 15.26	0.798
A′	77.98 ± 20.83	79.37 ± 17.12	0.459^a^	76.37 ± 14.22	76.49 ± 14.98	0.859
E′/A′	0.96 ± 0.28	0.98 ± 0.29	0.543	1.02 ± 0.26	1.02 ± 0.29	0.802
DT	201.22 ± 34.16	189.59 ± 26.13	<0.001	206.90 ± 27.62	204.45 ± 30.47	0.338
E mean	8.22 ± 1.42	8.49 ± 1.24	0.004	8.40 ± 1.37	8.46 ± 1.29	0.345
A mean	10.64 ± 2.89	10.88 ± 2.50	0.068	10.59 ± 2.30	10.56 ± 2.28	0.666
E_m_/A_m_ ratio	0.82 ± 0.27	0.90 ± 0.34	0.008	0.87 ± 0.27	0.89 ± 0.28	0.334
E/e′	9.18 ± 2.34	9.08 ± 2.18	0.733	8.88 ± 2.36	9.01 ± 2.18	0.740
Heart Rate, bpm	75.80 ± 12.60	73.68 ± 10.56	0.204	76.24 ± 12.72	74.56 ± 13.24	0.145
QT	376.76 ± 20.92	386.28 ± 26.27	0.037	371.31 ± 20.19	375.31 ± 21.21	0.014
Cor. *P* disp.	40.90 ± 10.13	32.23 ± 8.16	0.009	37.40 ± 7.89	35.26 ± 8.08	0.107
Cor. QT disp	41.63 ± 7.72	40.31± 7.01	0.196	36.49 ± 9.23	37.04 ± 8.97	0.145
Cor. Tp-e interval	81.52 ± 12.92	76.14 ± 7.77	0.008	76.06 ± 8.69	75.21 ± 8.69	0.631
cQT	421.47 ± 35.26	426.30 ± 26.09	0.337	420.75 ± 31.73	418.66 ± 30.38	0.485
Tp-E interval	376.76 ± 20.92	386.28 ± 26.27	0.037	371.31 ± 20.19	375.31 ± 21.21	0.014^a^
Tp-E/QT	0.19 ± 0.02	0.17 ± 0.03	0.315	0.16 ± 0.02	0.16 ± 0.02	0.588
Tp-E/cQT	0.17 ± 0.02	0.17 ± 0.02	0.944	0.17± 0.02	0.17 ± 0.02	0.851
RR interval	0.81 ± 0.12	0.83 ± 0.12	0.431	0.82 ± 0.14	0.83 ± 0.14	0.009

Data are presented as mean ± SD.

Parametric dependent samples were evaluated by paired samples T test.

A, mitral inflow contraction velocity; A_m_, atrial contraction wave using TDI; CDI, conventional Doppler imaging; DT, deceleration time; E, mitral velocity of early diastolic filling from transmitral flow; E_m_, early diastolic filling using TDI; E_m_/A_m_, diastolic myocardial motion velocities using TDI (early and late phase); IVRT, isovolumic relaxation time; TDI, tissue Doppler imaging; cPd, corrected *P* dispersion; cQT, corrected QT interval; cTp-e, corrected transmural dispersion of repolarization.

^a^ Friedman test was used for non-parametric dependent samples.

## 4. Discussion

This study's findings revealed significant improvements with ranolazine treatment in CSF patients compared to control subjects in diastolic flow parameters measured via IVRT and deceleration time. The negative effect of CSF on repolarization was demonstrated using novel repolarization parameters. The use of ranolazine caused significant improvements in some of these repolarization parameters in patients with CSF compared to the control subjects. The deterioration in diastolic parameters was previously demonstrated in patients with CSF in the literature based on repolarization findings assessed using echocardiography and electrocardiography. However, this study is the first study to date on the positive effects of ranolazine treatment in patients with CSF.

CSF is an important cause of angina pectoris. Its prevalence was reported to be between 0.2% and 24% (3). Noninvasive evaluation of CSF patients commonly performed due to ischemic symptoms usually reveals signs of ischemia, and therefore coronary angiography is performed. CSF is characterized by delayed progression of opaque material to the distal of the vessel without angiographically detectable stenosis. It is associated with atrial and ventricular arrhythmias, deterioration in systolic and diastolic functions, and even sudden death (10). Many studies have been published on the pathophysiology of CSF. These studies revealed microvascular and endothelial dysfunction as CSF's most widely accepted mechanism (11). Apart from epicardial arteries, small vessels with diameters of 400 micrometers or less are considered another risk factor. Additionally, endothelial dysfunction in microvascular structures reportedly increases resting vasomotor tone (12). CSF can cause ischemia at the microvascular level or acute coronary syndrome in severe cases. Diastolic functions are affected in myocardial tissues exposed to ischemia before systolic functions. For this reason, pulse and tissue Doppler echocardiographic parameters are very useful in the early prediction of subclinical ischemia.

Delayed epicardial flow in coronary arteries indicates worsening in diastolic parameters assessed by echocardiography. E/e′ is a parameter used to determine left ventricular diastolic pressures and is observed between 8 and 15 in series. E/e′ values above 15 indicate that the mean left ventricular diastolic pressure has increased abnormally. Mitral annular velocity (e′) is correlated with ventricular relaxation and increased preload. A study evaluating 48 patients with Doppler echocardiography and left ventricular relaxation found that end-diastolic pressures were impaired in patients with CSF (13).

Li et al. investigated conventional echocardiographic parameters in a total of 124 cases, including CSF patients, and determined that early diastolic mitral inflow velocity (E) and the ratio of early to late diastolic mitral inflow velocity (E/A) were significantly reduced in the CSF group compared to the control group. On the other hand, they did not find any significant difference between the groups in terms of IVCT, DT, IVRT, ejection time, and other conventional echocardiographic parameters (14). However, there is still no consensus in the literature on the effects of CSF on left ventricular systolic and diastolic functions. In one of these studies, Tanriverdi et al. found that diastolic functions have deteriorated, whereas systolic functions were maintained in CSF (15). In contrast, in a study by Zencir et al., conventional and tissue Doppler echocardiography did not reveal any significant effect of CSF (16). In comparison, in this study, conventional parameters were not changed significantly in the CSF group, yet mean Em/Am and E/A ratios were significantly lower in CSF patients compared to control subjects.

An increase in the cellular sodium level causes an increase in the intracellular calcium level. Calcium, which is already elevated after the end of systole and diastole, may place an extra load on the ventricular myocardium. It is hypothesized that ranolazine may improve myocardial functions by preventing the entry of sodium into the cell, reducing calcium accumulation in diastole as a result. Vincent et al. reported improvement in the diastolic parameters of patients using ranolazine indicated for stable angina pectoris. Ranolazine treatment reportedly improved deceleration, isovolumetric contraction, isovolumetric relaxation, myocardial performance index values, and ejection times (17). In a study conducted by Babalis et al. with 40 chronic coronary syndrome patients who were administered ranolazine for 3 months, the evaluation of diastolic functions with conventional echocardiography and tissue Doppler imaging revealed symptomatic recovery, as well as significant improvements in diastolic improvements, including E and A LV filling velocities, E/A ratio, DT, and IVRT however no changes in systolic functions (18). In comparison, in this study, a statistically insignificant improvement was observed in diastolic parameters with ranolazine treatment in both patient and control groups. Additionally, a statistically significant decrease in DT and IVRT and an increase in mean E/A ratio after ranolazine treatment in the patient group was observed, compared to the control group, where no statistically significant was observed. The positive effect of ranolazine treatment on diastolic functions has been shown in previous studies, with a more pronounced effect in patients with CSF than in patients without coronary artery disease.

Case reports and case series show that CSF is associated with arrhythmias and repolarization anomalies. Tp-e interval, Tp-e/QT ratio, and Tp-e/QTc ratio, newly defined repolarization parameters, are important predictors of ventricular arrhythmias (5). The QT segment and QTc on the ECG are associated with ventricular arrhythmias. QT interval dispersion indicates ventricular repolarization and cardiac electrical instability. It has been reported that QT interval dispersion may occur in the presence of myocardial ischemia and that its frequency further increases in the context of CSF (19). The T wave represents ventricular repolarization on the ECG. The Tp-e interval, the time elapsed between the peak and the end of the T wave, represents the total dispersion of ventricular repolarization between transmural, apicobasal, and global regions. Recent studies found that the Tp-e interval, Tp-e/QT, and Tp-e/QTc ratios were significantly longer in patients with CSF when compared to control subjects (20). These novel parameters provide more stable results considering the confounding effect of the heart rate. In comparison, in this study, an increase in corrected *P* and QT dispersion values was observed in patients with CSF compared to the control subjects. Additionally, the corrected Tp-e interval and Tp-e/QT ratio were found to be significantly higher in the CSF group than in the control group.

Ranolazine indirectly reduces intracellular calcium accumulation by inhibiting sodium entry into myocardial cells, relieving symptoms in patients with angina by lowering the oxygen consumption of myocardial cells. In addition to its anti-anginal features, ranolazine demonstrated a slight shortening or biphasic effect in M cells and caused prolonged epicardial cell action potentials in animal experiments. Thus, a concentration-dependent decrease in transmural action potential distribution was observed (21). Ranolazine also has an important anti-arrhythmic effect that inhibits early depolarization activity and transmural repolarization dispersion, preventing arrhythmias that may occur with increased QT duration (21).

The effects of ranolazine on the repolarization parameters, including Tp-e interval, Tp-e/QTc ratio, and *P* wave dispersion, were investigated in patients with stable coronary artery disease in the literature. In one of these studies conducted with 90 patients that were given ranolazine at various doses for one month and 85 control subjects, a significant decrease was observed in the Tp-e interval, Tp-e/QTc ratio, Tp-e/QT ratio, and *P*-wave dispersion in the ranolazine group (22). In a double-blind, placebo-controlled study, ranolazine decreased the T wave heterogeneity, representing repolarization in diabetic patients with non-flow limiting symptomatic coronary artery stenosis. Consequently, it was concluded that ranolazine alleviated the arrhythmias derived from ischemia (23). In comparison, in this study, a significant decrease was observed in the Tp-e interval, corrected *P* dispersion, and corrected Tp-e interval in the CSF group but not in the control group after ranolazine treatment. On the other hand, uncorrected QT value was significantly increased in both groups after ranolazine treatment. In addition, even though the use of ranolazine in patients with prolonged QT and with QT-prolonging drugs is contraindicated, ranolazine treatment did not result in pathologically prolonged QT.

Ranolazine, used for the treatment of chronic angina pectoris, reduces intra-cellular calcium levels by inhibiting voltage-gated sodium channels. It has an effect on repolarization parameters by prolonging the action potential duration on the myocardium. With the increase in clinical use, studies have shown that it is also effective on echocardiographic parameters. Summaries of large-scale studies on ranolazine other than our study are given in Table 3.

**Table 3:** Study summaries showing the effects of ranolazine on diastolic functions and repolarization parameters.

Author	Year	Aim	Treatment modality	Number of patients	Trial parameters	Results
Maier et al. (24)	2013	Diastolic functions in patients with HfpEF Inclusion criteria were EF ≥45%	Treatment consisted of intravenous infusion for 24 h	Patients were randomized to ranolazine (n: 12) and placebo (n: 8).	E/e′ ratio Repolarization measures as QT Interval in ECG	Echocardiographic diastolic parameters were not changed significantly. Non-significant QT interval increase was seen in ranolazine group.
Figueredo et al. (2)	2011	Improvement In left ventricular systolic and diastolic performance in patients with stable angina	Initial treatment was 500 mg bid. After 1 week dose increased to 1,000 mg bid. The evaluation was. Done minimum after 30 days.	22 patients without control group.	Mitral valve inflow velocities, tissue Doppler velocities, mitral annular diastolic velocities, E′/A′ ratio, E/E′ ratio, isovolumic contraction and relaxation times, ejection time and myocardial performance index	DT, IVCT, IVRT, ET and MPI were improved on ranolazine therapy.
Akcay et al. (22)	2021	Effects On ECG repolarization parameters	Patients used 750 mg or 1,000 mg doses. No significant difference was observed between the groups.	175 patients 90 of the m received ranolazine 85 of them was in control group.	Tp-e, QT, QTc intervals, Tp-e/QT, and Tp-e/QTc ratios	Tp-e interval, Tp-e/QT, Tp-e/QTc and PWD were significantly lower in the ranolazine group.
Babalis et.al (18)	2014	Effects Of ranolazine on left ventricular diastolic function in chronic coronart disease	Patients in ranolazine group used 500 mg bid ranolazine for 3 months.	40 pts; 20 in control group, 20 in ranolazine group	Mitral E velocity, mitral A velocity, E/A ratio, mitral anular e′ and a′ velocities, E/e′ ratio, DT, IVRT	Mitral E velocity, mitral A velocity, E/A ratio, e′, a′, E/e′, DT and IVRT significantly improved on ranolazine group.

### 4.1. Limitations of the study

Apart from its strengths, such as being the first study to date to reveal the positive effects of ranolazine treatment in patients with CSF, there were also some limitations to this study. First, the recommended maximum ranolazine dose (1,000 mg) could not be used since the study period was limited to 1 month. Longer follow-up with the inclusion of clinical adverse cardiovascular events may reveal further benefits of ranolazine in addition to its anti-anginal activity. The use of ECG due to cost and accessibility concerns rather than dynamic measurement in evaluating repolarization parameters may be considered a second limitation of this study. Another limitation of the study is the single-center recruitment of patients and the evaluation of ECG and echocardiographic examinations without blinding.

## 5. Conclusion

This is the first study on the effects of ranolazine treatment on diastolic function and repolarization parameters in patients with CSF. This study's findings reasserted the adverse effects of CSF on diastolic functions and repolarization parameters and demonstrated that ranolazine positively affected both the impaired diastolic functions evaluated by classical echocardiographic methods and repolarization parameters, particularly in patients with CSF, compared to the control group. In addition, while causing improvements in ECG parameters, ranolazine did not induce any pathological QT abnormality. Further large-scale studies with more extended follow-up durations are needed to corroborate the findings of this study.

## Data Availability

The raw data supporting the conclusions of this article will be made available by the authors, without undue reservation.
